# Toward Robust Lung Cancer Diagnosis: Integrating Multiple CT Datasets, Curriculum Learning, and Explainable AI

**DOI:** 10.3390/diagnostics15010001

**Published:** 2024-12-24

**Authors:** Amira Bouamrane, Makhlouf Derdour, Akram Bennour, Taiseer Abdalla Elfadil Eisa, Abdel-Hamid M. Emara, Mohammed Al-Sarem, Neesrin Ali Kurdi

**Affiliations:** 1 LIAOA Laboratory, University of Oum El-Bouaghi-Larbi Benmhidi, Oum El-Bouaghi 04000, Algeria; amira.bouamrane@univ-oeb.dz (A.B.); derdour.makhlouf@univ-oeb.dz (M.D.); 2 LAMIS Laboratory, Echahid Cheikh Larbi Tebessi University, Tebessa 12002, Algeria; 3 Applied College, King Khalid University, Mahayil 62529, Saudi Arabia; teisa@kku.edu.sa; 4 Department of Computers and Systems Engineering, Faculty of Engineering, Al-Azhar University, Cairo 11884, Egypt; abdemara@azhar.edu.eg; 5 Department of Information Technology, Aylol University College, Yarim 547, Yemen; mohsarem@gmail.com; 6 College of Computer Science and Engineering, Taibah University, Medina 41477, Saudi Arabia; nkordi@taibahu.edu.sa

**Keywords:** CT scan, pulmonary nodules, DL, diagnosis, XAI, curriculum learning, mixup

## Abstract

**Background and Objectives:** Computer-aided diagnostic systems have achieved remarkable success in the medical field, particularly in diagnosing malignant tumors, and have done so at a rapid pace. However, the generalizability of the results remains a challenge for researchers and decreases the credibility of these models, which represents a point of criticism by physicians and specialists, especially given the sensitivity of the field. This study proposes a novel model based on deep learning to enhance lung cancer diagnosis quality, understandability, and generalizability. **Methods:** The proposed approach uses five computed tomography (CT) datasets to assess diversity and heterogeneity. Moreover, the mixup augmentation technique was adopted to facilitate the reliance on salient characteristics by combining features and CT scan labels from datasets to reduce their biases and subjectivity, thus improving the model’s generalization ability and enhancing its robustness. Curriculum learning was used to train the model, starting with simple sets to learn complicated ones quickly. **Results:** The proposed approach achieved promising results, with an accuracy of 99.38%; precision, specificity, and area under the curve (AUC) of 100%; sensitivity of 98.76%; and F1-score of 99.37%. Additionally, it scored a 00% false positive rate and only a 1.23% false negative rate. An external dataset was used to further validate the proposed method’s effectiveness. The proposed approach achieved optimal results of 100% in all metrics, with 00% false positive and false negative rates. Finally, explainable artificial intelligence (XAI) using Gradient-weighted Class Activation Mapping (Grad-CAM) was employed to better understand the model. **Conclusions:** This research proposes a robust and interpretable model for lung cancer diagnostics with improved generalizability and validity. Incorporating mixup and curriculum training supported by several datasets underlines its promise for employment as a diagnostic device in the medical industry.

## 1. Introduction

**Figure 1:**
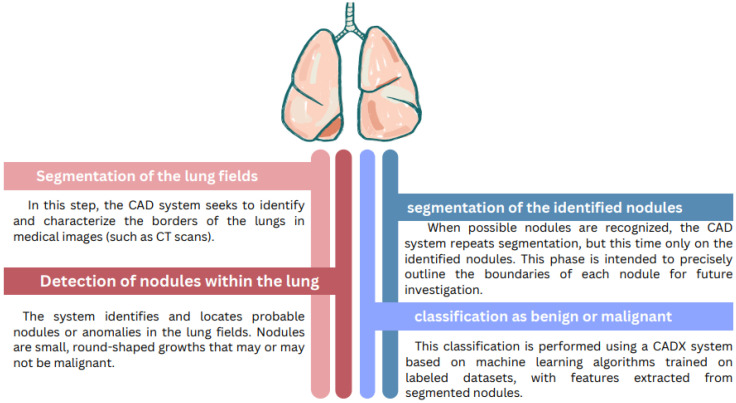
CADx Phases.

Given the success of artificial intelligence in general and deep learning in particular in the health field and for disease diagnosis, researchers have increasingly focused on developing computer-aided diagnosis (CADx) systems [1]. Lung cancer has been a prominent area of focus for these systems due to the severity of this disease [2]. It ranks as the top cancer type and is the leading cause of cancer-related deaths worldwide, with more than 2 million cases estimated in 2024 [3], according to the World Health Organization and the American Cancer Society, mainly due to delayed diagnoses. Research has revealed that timely detection is a key factor contributing to significantly improving five-year survival rates and treatment success [4]. This has led researchers to investigate ways in which CADx could further be developed to improve precision and timeliness, especially considering the scarcity of qualified specialist personnel, human subjectivity, and the exhaustion of radiologists due to extended working hours [5,6]. This disease occurs because of the unregulated growth of cells in the body, mostly seen in the lung parenchyma, and it can start anywhere but most often does so from the epithelial tissues within the bronchi or alveoli [7]. Nevertheless, it is, more often than not, manageable, provided that it is detected early enough [8]. The actual threat of lung cancer becomes substantial when metastasis takes place [9]. The growth and dispersion of lung nodules vary with the type and features of lung cancer, and are commonly classified into two main types: small-cell lung carcinoma and non-small-cell lung carcinoma [10]. The computed tomography (CT) scan is a modality that has been around for several years and is mainly used in lung cancer screening [11]. It employs several X-rays, which are later manipulated using computer technology in order to produce a 3-D image of a particular region of interest [12]. It provides a number of cross-sectional images that can identify masses as small as 1 to 2 millimeters in size [13]. However, CT scans have some drawbacks, including reduced contrast for soft tissues [14], particularly in the case of blood-borne tumor infiltration into soft tissue, and the risk of artifacts created from varying tumor shapes and the extents of growth [15]. Additionally, CADx allows for the processing of CT images, recognizes different features in the images, removes artifacts, and analyzes details and characteristics such as the tumor’s shape, size, and location [16]. Moreover, CT images are processed using deep learning algorithms, which help extract high-quality features from CT scans and aid tumor classification [17]. Moreover, the process of lung tumor CADx contains four phases [18], starting with segmenting the lungs from the surrounding tissue and lung wall. Next, the nodules are detected and segmented. We distinguish various segmentation techniques, such as Mask R-CNN, thresholding, U-Net [19], graph-based methods, and DeepLabv3 [20]. After segmentation, the final step was to classify these nodules as either benign or malignant (Figure 1).

Convolutional neural networks (CNNs) enable CT image analysis to extract high-quality and quantitative features through a hierarchical representation of input data [21]. Additionally, transfer learning and deep models trained on large datasets can learn a substantial number of features, making them highly effective for extracting extensive features to distinguish the characteristics of these tumors [22]; such models include EfficientNet [23], MobileNet, DenseNet [24], VGG16 [25], VGG19 [26], and ResNet50 [24]. Moreover, incorporating DL models into hybrid models further augments the feature extraction capability [27,28]. Considering the significance of data, numerous medical centers and specialists have made available lung cancer databases to assist researchers in creating diagnostic systems such as LIDC-IDRI [29,30], NIH dataset [31], IQ-OTH/NCCD [32], and LUNA-16 [33]. Nevertheless, this domain suffers from data heterogeneity and inaccessibility [34]. To solve this problem, some researchers have employed techniques such as data augmentation [35] or dataset fusion [36].

This study presents a hybrid model for lung cancer diagnosis that combines the effectiveness of MobileNetV3Small and ResNet50 for feature extraction and is based on five different and heterogeneous datasets to avoid bias. The proposed approach employs curriculum learning to train on these datasets from the simplest to the most complex data and also uses mixup augmentation techniques to enhance the robustness of the model and improve its generalization capability. Finally, the Gram-CAM is applied to improve model interpretability and explainability by identifying the key features on which the model bases its decisions. The primary contributions of this study are as follows:

### Research Contributions

- A new model that adopts curriculum strategies to analyze training datasets, training on data from the easiest to the most complex, which improves learning efficiency and enables the understanding of harder tasks.
- High-quality and abundant features were obtained using ResNet50 and MobileNetV3 Small with reduced complexity, as they are used solely for feature extraction.
- Data augmentation and model robustness were enhanced using mixup augmentation techniques by randomly merging images with their labels. This allows the model to analyze more images with diverse features, thereby increasing its robustness and generalization ability.
- Bias reduction was achieved by using multiple datasets, and five different databases were used to train the model with diverse demographic samples and varying characteristics, reducing the model’s data dependency and bias, and enhancing its effectiveness.
- Generalizability Assessment: by evaluating the model using a totally external dataset.
- The model’s explainability and trustworthiness are enhanced by using Grad- CAM to identify the most relevant regions in the screening that the model relied on for its decisions, thereby increasing the model’s transparency.

The rest of the paper is organized as follows. The Section 2 analyzes related works connected to the paper’s research, focusing on using CT scans and deep learning. The Section 3 presents the materials and methods, summarizing the proposed model, methods used, research problem, desired objectives, and various databases used. The results are presented in the Section 4. The impacts of using curriculum learning and mixup methods are analyzed, and explainable AI is used to rationalize some of the classifications made by the model. Finally, the Section 5 reviews the contents of this paper, and some areas of research that could expand the results are proposed.

## 2. Related Work

Many diagnostic systems have utilized CNNs and deep learning to diagnose lung cancer and have proven to be effective. A number of them used LIDC-IDRI to evaluate their models, such as Zhao et al., who developed a new transformer-inspired model for lung nodule classification based on the LIDC-IDRI dataset; the BiCFormer enhanced feature extraction using a novel multi-layer GAN for data augmentation and a bi-level coordinate (BiC) encoder. This model achieved 97.4% of accuracy [37]. Similarly, Gopinath et al. presented a novel classification method, Deep Fused Features-Based Cat-Optimized Networks (DFF-CON) based on LIDC-IDRI to enhance lung cancer diagnosis from CT scans, and they employed a saliency map to highlight important features. DFF-CON achieved high performance across metrics, with 99.89% accuracy and 99.88% F1-score, outperforming existing models [38]. Saied et al. developed AI approaches to classify pulmonary nodules efficiently from CT scans. Using DenseNet-121 and SVM based on the LIDC-IDRI dataset, they focused on extracting features and then applied PCA for feature selection, and DenseNet-121 achieved the best results combined with SVM. They also tried VGG-16 and VGG-19, and DenseNet-169. DenseNet-121 combined with SVM achieved an accuracy of 90.39%, sensitivity of 90.32%, and specificity of 93.65% [39]. Meng et al. proposed a machine learning model using CT features and serum biomarkers based on two local datasets by highlighting nodule diameter and average CT value as key predictive features, aiding early intervention and improving early lung cancer diagnosis. The Gradient Boosting Machine (GBM) model showed superior accuracy and AUC of 99% and 93.1%, respectively, with 85.7% and 95.5% in the external phase. The study also included the use of Shapley additive explanations (SHAP) to explain the features on which the model’s decision is based [40]. Utilizing the Kaggle CT scan of lung cancers, Lanjewar et al. suggested a modified DenseNet201 architecture to classify the four types of lung cancer. They conducted five experimental scenarios, including one-upping the architecture of DenseNet201 in a bid to make it less trainable and also using it as a feature extractor with a Support Vector Machine, Logistic Regression, Random Forest, Decision Tree, Gaussian Naive Bayes, and KNN. Two performance improvement techniques were used to achieve a feasible number of extracted features, ETC and MRMR. The method proposed achieved good results, with the highest accuracy of 100%, an AUC value of 99.25%, and a Kappa score of 93%. It should be mentioned that 5-fold cross-validation achieves an accuracy level of 95%. However, despite these promising results, the authors stated that there are limitations, such as the small dataset and the necessity to validate and further test on larger datasets, for the model’s improvement in robustness and generalizability [41]. Shen et al. proposed a WS-LungNet based on the LIDC-IDRI dataset, a weakly supervised deep learning framework to segment 3D pulmonary nodules based on unlabeled data and to address scarcity and inconsistency in computer-aided lung cancer diagnosis (CAD) from CT images. By using semi-supervised segmentation with adversarial learning and cross-nodule attention mechanisms, WS-LungNet achieved 82.99% competition performance metric (CPM), an 88.63% area under the curve (AUC), and DROC of 87.12% [42].

Wahab et al. presented a deep learning-based model for lung cancer detection using DenseNet-121 CNN for feature extraction, deep autoencoders to minimize the feature dimensionality, and MobileNetV3-Small for classification. The proposed approach was evaluated using the lung-PET-CT-Dx dataset, and achieved 98.6% accuracy with reduced computational requirements. They employed techniques such as quantization-aware training and early stopping to optimize the performance. Despite this performance, the authors declared that there is a lack of highly balanced datasets, and the dataset used was of low-quality images [43]. On the contrary, in [44], the authors developed Lung-EffNet, a transfer learning-based model using EfficientNet variants for accurate lung cancer classification from the IQ-OTH/NCCD dataset augmented using the GAN technique; the EfficientNetB1-based Lung-EffNet achieved 99.1% accuracy and a score of 97% to 99% of ROC. The model outperformed other CNNs, offering efficiency and lower training demands, making it a promising tool for clinical deployment. They also employed a Grad-cam to assess model interpretability. Despite this performance, the dataset used is very small, even with data augmentation; the model suffers from data dependency, which limits its generalizability, as shown in Table 1. 

Despite the remarkable results achieved by previous studies, most of these models have been evaluated for their effectiveness using only a single dataset. This can lead to a certain level of data dependency, especially because these datasets are homogeneous, gathered from the same demographic sample, and use the same imaging equipment. Consequently, the models have a narrow range of characteristics they can identify, making them even more non-generalizable. However, most previous research has only tested such models on the available test samples from the same database and never assessed the models on external demographic factors to determine their generalizability. Furthermore, while deep learning models are effective and accurate, they are considered “black boxes”, as the processes leading to their classifications are uninterpretable. Owing to the existing circumstances, there is a feeling of uneasiness among practitioners, particularly considering the nature of the undertaking. Many previous studies have not been sufficient in this area of work, and a large gap remains. Table 1 summarizes the related work.

## 3. Materials and Methods

### 3.1. Problem Statement

Physicians and radiologists require a lung cancer detection system that can accurately screen patients in different cases [45] and minimize false positive and false negative rates [46]. In addition, the system should be sufficiently reliable to explain their results [47] as an additional support; hence, it functions as a dependable second opinion.

### 3.2. Research Objectives

This study aims to achieve a set of objectives, which are as follows:

- To explore a new lung cancer CADx.
- To reduce data dependency and bias by using five training datasets.
- To improve the model’s robustness and generalization capability by employing a mixup augmentation technique and curriculum learning strategy.
- To evaluate the model generalizability using an external dataset.
- To enhance trustworthiness and interpretability.

### 3.3. Datasets and Preprocessing

**Figure 2:**
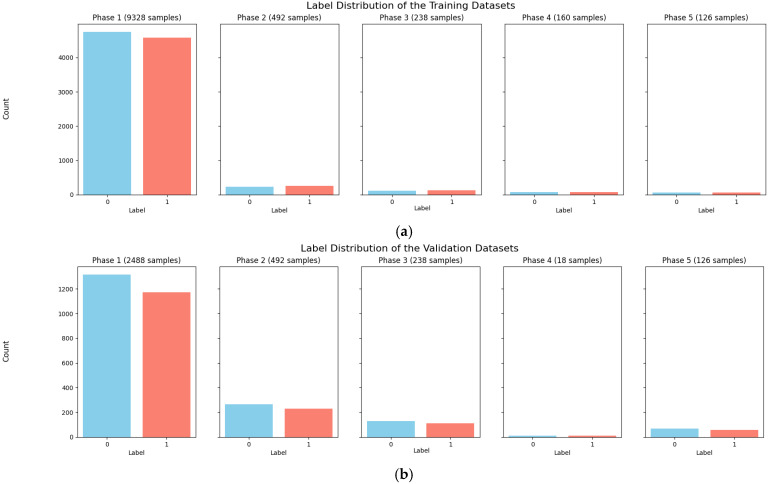
Dataset distribution: training and validation. (**a**) Training; (**b**) Validation.

It is worth mentioning that all datasets used in this work are publicly available. We used a subset of the LIDC-IDRI dataset along with four other datasets for training and validation of the model and an internal test [29,48,49,50,51]. The preprocessing included converting images to grayscale and resizing them to 224 × 224-pixel dimensions to fit the model input shape and reduce image complexity while keeping important information. The first three datasets were subjected to primary data augmentation using oversampling to eliminate class bias and were then split into training and validation datasets. Downsampling was used for the fourth and fifth datasets, with the data being split into training, validation, and testing datasets. This ensured that the model in the internal test would not view the test scans during training or validation. The resulting splits were as follows: 9328, 492, and 238 images were used for training from the first three datasets. For validation, we used 2488 images from the first dataset and 492 and 238 images from the other two datasets. For the fourth and fifth datasets, 160 and 126 images were used for training, 18 and 126 for validation, and 162 were reserved for testing from these two datasets, as shown in Figure 2.

To further evaluate the generalizability of the model, we tested it on a completely unseen external dataset, the Lung Cancer CT (LDCT) dataset [52].

### 3.4. The Utilized Techniques and Proposed Methodology

#### 3.4.1. MobileNetV3 Small

MobileNetV3 Small is a lightweight CNN that is designed for resource-efficient mobile applications. It incorporates concepts from MobileNetV2, such as depthwise separable convolutions, while introducing new elements, such as squeeze-and-excitation modules and neural architecture search to optimize the network’s structure. This model is ideal for achieving a balance between performance and computational efficiency in tasks like image classification and object detection on devices with limited resources [53].

#### 3.4.2. Resnet50

ResNet50 is a deep residual network architecture comprising 50 layers, and it is well known for introducing residual learning, which helps in training very deep networks by mitigating the vanishing gradient problem. The model uses identity mappings to add shortcut connections between layers, facilitating efficient backpropagation and improving model convergence. ResNet-50 is widely used in various computer vision tasks, such as image recognition, object detection, and feature extraction, owing to its high accuracy and performance on large datasets like ImageNet [54].

#### 3.4.3. Classification Layers

**Figure 3:**
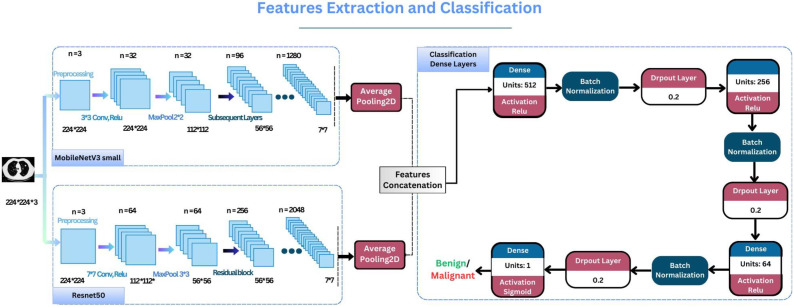
Feature extraction and classification dense layers.

The categorization step includes three dense layers (using ReLU activation) within ‘l2’ normalization, each one of them followed by batch normalization, then dropout (20%) to avoid overfitting, and finally, the output layer with one unit and using the sigmoid function. Figure 3 illustrates the feature extraction stage using both MobileNetV3Small and ResNet50 and the classification phase using dense layers.

#### 3.4.4. Mixup Methods

**Figure 4:**
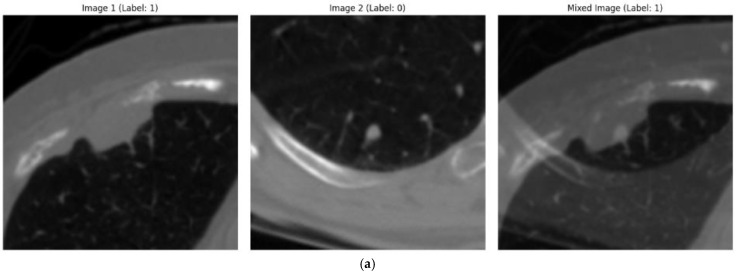

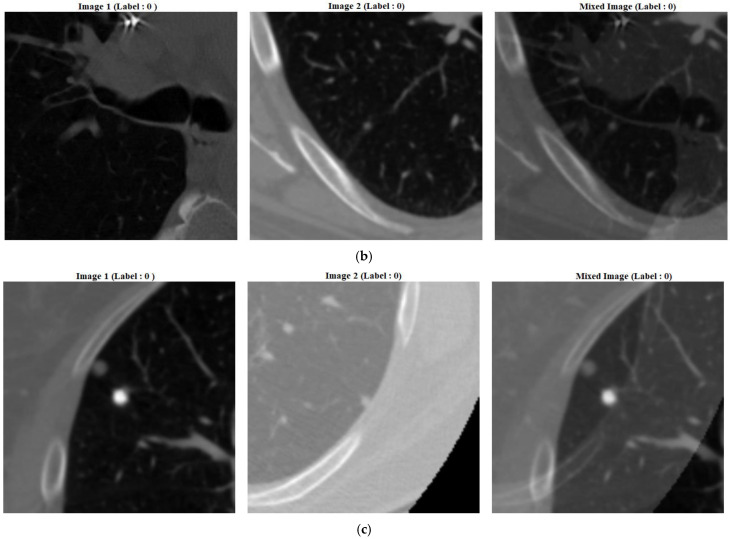
Visualization of mixup augmentation on image samples. The three subfigures (**a**–**c**) represent the application of mixup augmentation. Each subfigure contains two images, both annotated with labels by radiologists. The third image in each subfigure shows the result of applying the mixup augmentation technique to these two images and their corresponding labels.

Mixup is a data augmentation technique that blends pairs of input data and their labels to create new synthetic samples. This reduces overfitting, improves model robustness, and enhances generalization to unseen data by producing smoother decision boundaries. Mixup has been effective across various domains, boosting model performance in tasks such as image recognition and natural language processing [55]. Figure 4 represents the application of mixup to random images from the used dataset.

#### 3.4.5. Curriculum Learning

Curriculum learning is a training strategy in which the model is gradually exposed to increasingly complex data, mimicking how humans learn. The approach begins with simpler examples and progressively introduces harder examples, allowing the model to develop more reliable and stable representations. It has been applied to fields such as language modeling, image recognition, and reinforcement learning, demonstrating improvements in training speed, convergence, and overall performance [56].

#### 3.4.6. Grad-CAM

Gradient-weighted Class Activation Mapping (Grad-CAM) was proposed by Selvaraju et al. to produce visual explanations of the decision-making process in convolutional neural networks. It leverages the gradient information from the last convolutional layer, which typically strikes the best balance between high-level semantics and detailed spatial features. The importance of each neuron in the model’s decision making is assessed. Grad-CAM generates a heatmap that highlights the regions of the input image that most influence the model’s prediction, helping to make the typically opaque nature of CNNs more understandable and interpretable [57].

### 3.5. The Proposed Methodology

**Figure 5:**
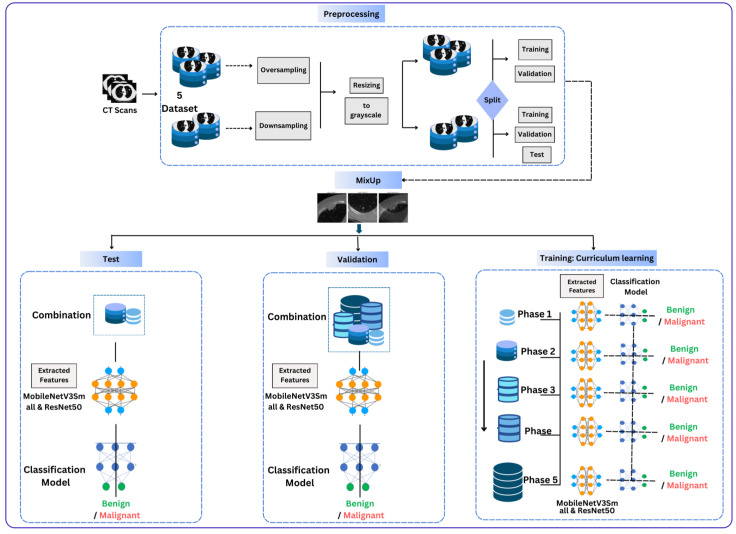
Proposed approach: a simplified illustration of the model.

The solution proposed in this work can be used to diagnose lung cancer with high efficiency and accuracy and with considerable generalizability. It employs several distinct CT datasets, making it accessible to the model to eliminate data dependence and bias. This strategy presents a hybrid feature extraction model that integrates MobileNetV3Small and ResNet50. In addition, data augmentation is employed using a mixup, thereby making the model more robust and improving the generalization performance. Curriculum learning mimics the human learning process by training the model using simple and complex patterns. Finally, the Grad-CAM technique is applied to the proposed model to improve its comprehensibility and reliability, as shown in Figure 5.

### 3.6. Pseudocode for Mixup Augmentation and Training Phase Using Curriculum Learning

To enhance the reusability of the model, this section includes the pseudocode of the three main techniques used in the proposed model.

- Mixup augmentation (Algorithm 1): This method generates an image and labels forged together by combining two images and their respective labels using a random interpolation factor (*λ*) sampled from a Beta distribution.
- Mixup data generator (Algorithm 2): Augmented data batches are constructed by mixing augmented input sample batches, which creates an infinite variety of fresh augmented datasets in training.
- Training with mixup augmentation (Algorithm 3): In the training phase, curriculum earning was adopted. During each of the five phases, data and labels were processed to create augmented batches. The model was trained for 100 epochs per phase, with callbacks such as learning rate reduction and early stopping. The training procedure was monitored by updating the total number of epochs and providing training history records.

**Algorithm 1:** The pseudocode of Mixup Augmentation Method (MixupAug)(1) procedure MixupAugmentation(image1, image2, label1, label2, alpha) (2) λ ⟵ RandomBeta(alpha, alpha) // generate random lambda parameter (3) MixedImage ⟵ λ * image1 + (1 - λ) * image2 // perform mixup on images (4) MixedLabel ⟵ λ * label1 + (1 - λ) * label2 // perform mixup on labels (5) Result ⟵ Clip(MixedLabel, 0, 1) // clip mixed label values (6) Return(MixedImage, Result) // return mixed image and label end procedure
**Algorithm 2:** The pseudocode of Mixup Data Generator (MixupGen)(1) procedure MixupDataGen(x_data, y_data, batch_size, alpha) (2)   While(True) // start infinite loop for generating batches (3)      Shuffle(indices) // shuffle the data indices (4)      For(i ϵ Range(0, len(x_data), batch_size)) // iterate over data batches (5)            x_batch, y_batch ⟵ CurrentBatch(x_data, y_data, indices, i,                     batch_size) // get the current batch (6)            AugBatch ⟵ ApplyMixupToBatch(x_batch, y_batch, alpha) //                                apply mixup augmentation (7)            Shuffle(AugBatch) // shuffle the augmented batch (8)            Return(AugBatch) // return the augmented batch end procedure
**Algorithm 3:** Training Model with Mixup Augmentation (TrainMixup)(1) procedure TrainMixup(datasets, model, batch_size, epochs, callbacks) (2)       For (x_data, y_data, phase_name) ϵ datasets // iterate through datasets (3)            TrainGen ⟵ MixupDataGen(x_data, y_data, batch_size,                   alpha=0.01) // initialize mixup generator (4)            Steps ⟵ ComputeSteps(len(x_data)) // compute steps per epoch (5)           TrainModel(model, TrainGen, epochs, Steps, callbacks)// train model  


## 4. Results and Discussion

The training of the model was performed using five different datasets, beginning with a high-resolution dataset (LIDC-IDRI), and the remaining four datasets were given to the model in order. In addition, the Adam optimizer was used with a learning rate of 0.01 and a batch size of 32 suitable for all five datasets, and ReduceLROnPlateau was used to reduce the learning rate when the performance of the model did not increase for a while.

**Figure 6:**
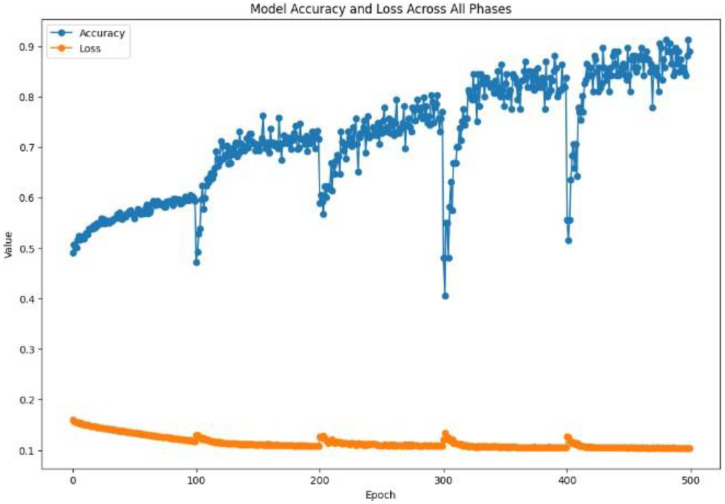
Training performance: loss and accuracy.

**Figure 7:**
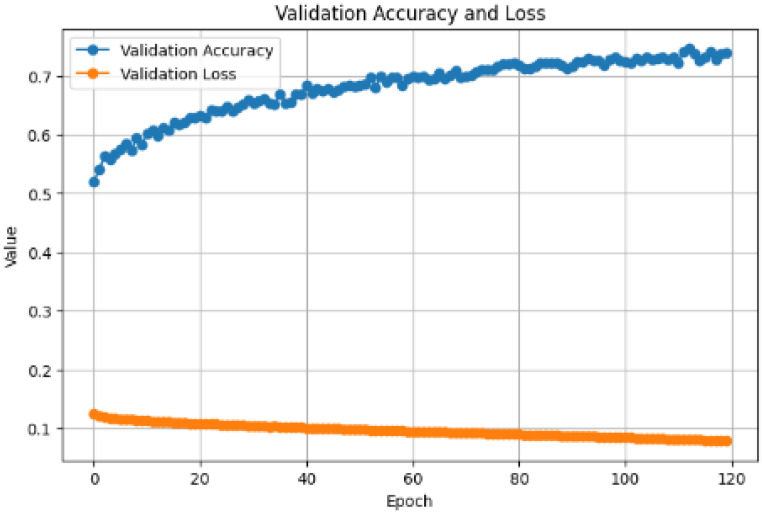
Validation performance: loss and accuracy.

**Figure 8:**
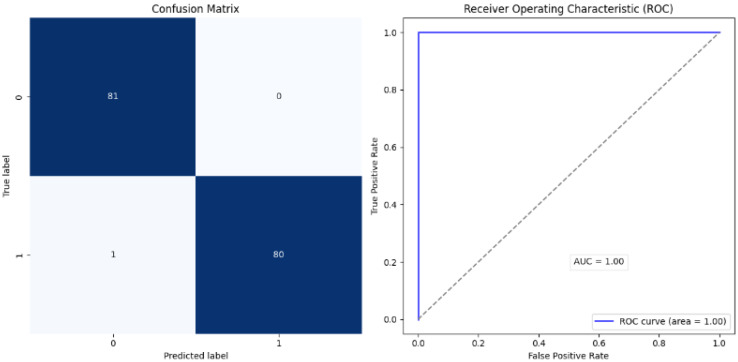
Confusion matrix: internal test.

The proposed approach achieved comparable results. Figure 6 and Figure 7 show the accuracy and loss curves during training and validation. The mixup technique made the model somewhat heavy. It was noted that when the model was supplied with a new dataset each time, the accuracy decreased by approximately 40% because the new samples were unfamiliar to the model. However, the model adapted quickly and improved its performance. The same was observed for the loss, where only a very small change occurred, which was soon corrected. Moreover, the model achieved excellent results during testing, with an accuracy of 99.38%, precision and specificity of 100%, sensitivity of 98.76%, an F1-score of 99.37%, and 100% AUC and ROC. The analysis in Table 2 and Figure 8 shows the confusion matrix of the proposed model for the testing stage and proves that its performance surpasses the majority of the models available in the literature, with the exception of the model of Gopinath et al., which achieved an accuracy of 99.89%, and F1-score, sensitivity, and precision of 99.8% using DCNN. However, the authors used only one dataset, the LIDC-IDRI dataset, without any augmentation. Therefore, the model could be considered biased toward this dataset, particularly because it has a high resolution and is homogeneous [38]. In addition, the proposed model by Lanjewar et al. stated that their proposed model reached 100% accuracy with an AUC of 99.25%. Despite this high performance, the results obtained in the model testing only included data from the same demographic sample, meaning that the studied features were similar. This creates a problem and challenge in terms of generalizability [41].

**Figure 9:**
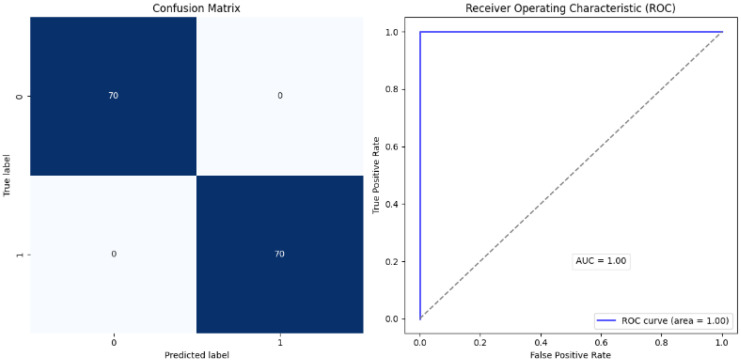
Confusion matrix: external test.

**Figure 10:**
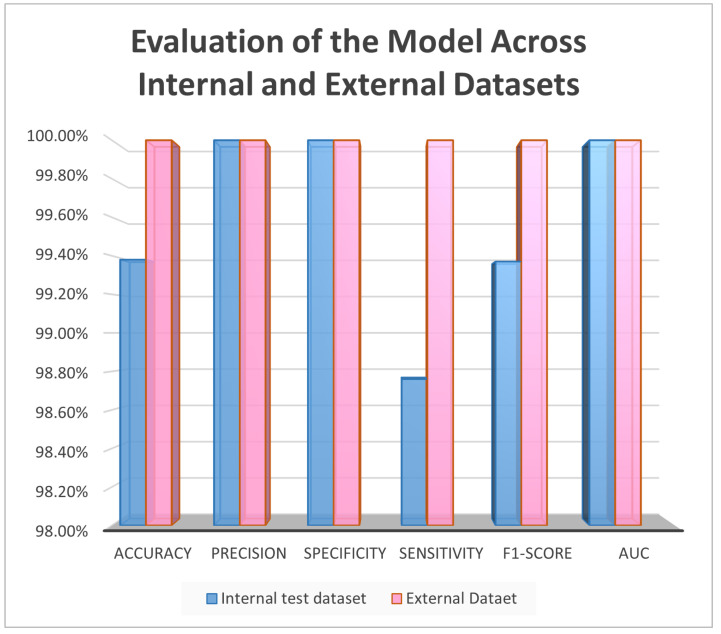
Testing of the model across internal and external datasets.

**Figure 11:**
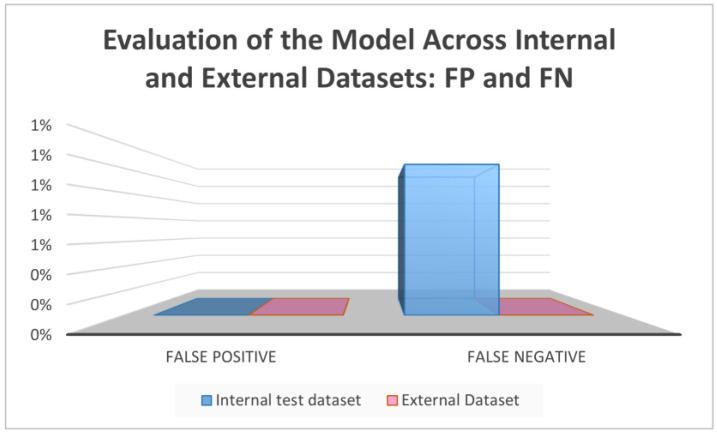
Testing of the model across internal and external datasets: FP and FN.

However, it should be mentioned that such models have been trained and evaluated using only a single dataset, which introduces some bias and data dependence. On the contrary, the proposed model in this paper performed well not only on the internal training and testing datasets but also on entirely different external datasets, shown in Table 2 and Figure 9, where 100% was registered across all metrics, as well as 0% false negative and false negative rates, indicating excellent generalization potential. Furthermore, as shown in Figure 10 and Figure 11, the performance of the assessed model was consistent across internal and external datasets. However, additional tests should be performed using additional external datasets that vary in nature. Additionally, the complexity of the model during training and validation still requires further experimentation to enhance the speed while lowering the complexity. Other methods, such as CutMix or Cutout, may be more effective.

**Figure 12:**
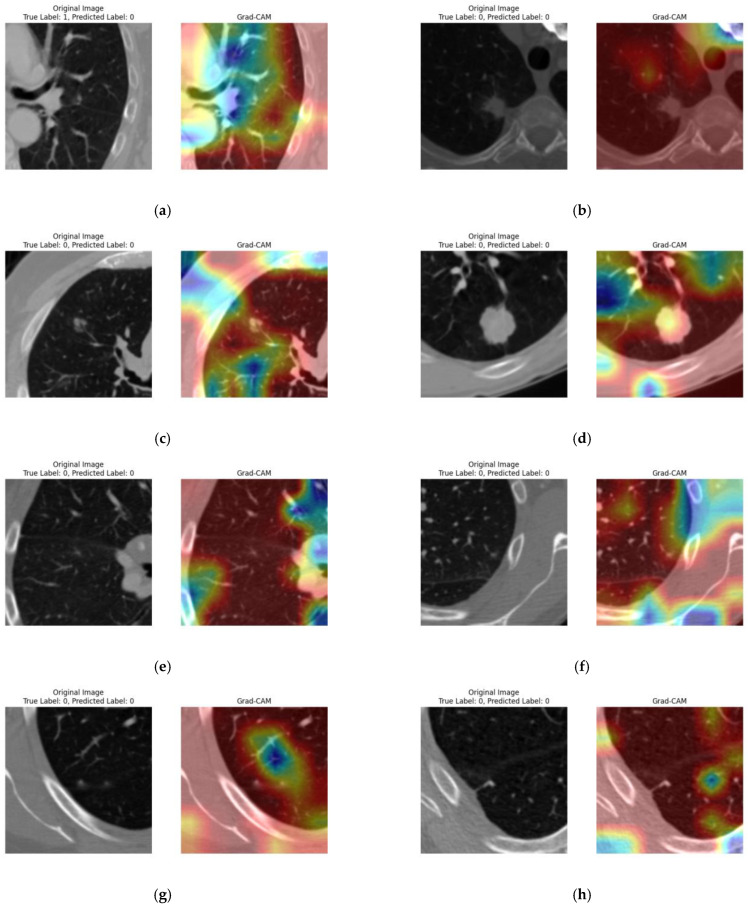
GRAD-CAM heatmaps: visualizing model attention in predictions. The eight subfigures (**a**–**h**) represent the original image with its true label and the predicted label by the proposed model. The second part of each subfigure shows the Grad-CAM image, which highlights the important features that the model focuses on during prediction.

To improve the model’s reliability and interpretability, the Grad-CAM technique was used with an alpha value of 0.1, as shown in Figure 12. Eight images were randomly selected to visualize the features and areas the model focused on when making its classifications. In one instance, the model incorrectly classified an image as benign, as shown in Figure 12a; the model’s attention was primarily on the outer wall of the lungs, excluding the tumor region. In contrast, the model correctly classified the remaining images, concentrating on specific areas along the lung wall and on impurities within the images, as highlighted by the color intensities.

## 5. Conclusions

This study provides a model for diagnosing lung cancer that is interpretable, robust, and accurate with good generalization. The proposed model used a hybrid combination. 

MobileNetV3Small and ResNet50 models were used for feature extraction and attention was injected into the dense layers for classification. To enhance the strength of the model, we used mixup augmentation based on the idea of overlaying the two images and their labels. Furthermore, the model adopted a curriculum learning paradigm during training for all five datasets. The results were excellent, with 99.08% accuracy, 0% false positive rate, 1.23% false negative rate, and 100% in ROC, AUC, precision, and specificity scores. An entirely external dataset was used to assess the model’s generalization ability, achieving 100% across all metrics with no false positives or negatives. Furthermore, GRAM-CAM was applied to enhance the model’s trustworthiness and comprehensibility. Although the results obtained are commendable, additional experiments are still needed. In the future, we will seek to experiment with different augmentation techniques, such as CutMix, Cutout, or RandAugment, which could improve the model’s performance and generalization ability. In addition, testing the proposed model on a broader range of external datasets with diverse demographic characteristics is crucial. The model complexity experienced during the training phase remains a myth that must be alleviated by optimizing the model, which will also be explored. Moreover, including multi-modal data in a curriculum learning approach could allow for stepwise learning of different imaging datasets. Therefore, the generalization of the model trained on different datasets from various imaging modalities will be explored.

## Figures and Tables

**Table 1:** Related work comparison.

#	Study	Year	Approach	Dataset	Strengths	Limitation
01	Zhao et al. [37]	2024	BiCFormer	LIDC-IDRI	Accuracy = 97.4%	Lack of interpretability, homogenous dataset, limited dataset size
02	Meng et al. [40]	2024	Gradient Boosting Machine	Local hospital datasets	Accuracy = 99%, AUC = 93.1%, External validation: Accuracy = 85.7%, AUC = 95.5%	Homogenous dataset
03	Gopinath et al. [38]	2023	DFF-CON using DCNN	LIDC-IDRI	Accuracy = 99.89%, F1-score = 99.88, Sensitivity = 99.8%, Specificity = 99.76%, Precision = 99.8%	Limited dataset size, homogeneity, potential for bias, limited reliability
04	Saied et al. [39]	2023	DenseNet-121 and SVM	LIDC-IDRI	Accuracy = 90.39%, Sensitivity = 90.32%, Specificity = 93.65%	Small, homogenous dataset, potential overfitting, limited generalizability
05	Lanjewar et al. [41]	2023	SVM, LR, RF, DT, GNB, KNN	Chest-CT Kaggle dataset	Accuracy = 100%, AUC = 99.25%, Kappa = 93%	Limited generalizability, feature dependency, limited interpretability
06	Wahab et al. [43]	2023	DenseNet-121 and MobileNetV3-Small	Lung-PET-CT-Dx dataset	Accuracy = 98.6%, Precision = 97.9%, Recall = 98.1%, F1-Score = 98, Kappa = 95.8%	Imbalanced dataset, limited interpretability
07	Raza et al. [44]	2023	EfficientNetB1-based Lung-EffNet	IQ-OTH/NCCD	Accuracy = 99.10%, Precision = 99.22%, Recall = 97.22%, F1score = 98.16%	Very small, homogenous dataset
08	Shen et al. [42]	2023	WS-LungNet	LIDC-IDRI	CPM = 82.99%, AUC = 88.63%, DROC = 87.12%	Lack of interpretability, small and homogenous dataset

**Table 2:** Model results across the internal and external datasets.

Proposed Model	Accuracy	Precision	Specificity	Sensitivity	F1-Score	AUC	False Positive	False Negative
Internal test dataset	99.38%	100%	100%	98.76%	99.37%	100%	00%	1.23%
External Dataset	100%	100%	100%	100%	100%	100%	00%	00%

## Data Availability

The data supporting the findings of this study are based on publicly available datasets in the following repositories:
LIDC-IDRI dataset at https://paperswithcode.com/dataset/lidc-idri (accessed on 14 June 2023).
CT Scan Images for Lung Cancer [49] at: https://www.kaggle.com/datasets/dishantrathi20/ct-scan-images-for-lung-cancer Lung Cancer Dataset [50] at: https://www.kaggle.com/datasets/jayaprakashpondy/lung-cancer-dataset Chest CT-Scan images Dataset [51] at: https://www.kaggle.com/datasets/mohamedhanyyy/chest-ctscan-images DLCTLUNGDetectNet-Lung Tumor Dataset [52] at: https://www.kaggle.com/datasets/harshaldharpure/dlctlungdetectnet-lung-tumor-dataset.
